# Effect of Hormone Replacement Therapy on Bone Mineral Density and Body Composition in Chinese Adolescent and Young Adult Turner Syndrome Patients

**DOI:** 10.3389/fendo.2019.00377

**Published:** 2019-06-12

**Authors:** Li Li, Xiu Qiu, Gendie E. Lash, Lianxiong Yuan, Zichao Liang, Li Liu

**Affiliations:** ^1^Department of Obstetrics and Gynecology, Guangzhou Women and Children's Medical Center, Guangzhou Medical University, Guangzhou, China; ^2^Division of Birth Cohort Study, Guangzhou Women and Children's Medical Center, Guangzhou Medical University, Guangzhou, China; ^3^Guangzhou Institute of Pediatrics, Guangzhou Women and Children's Medical Center, Guangzhou Medical University, Guangzhou, China; ^4^Department of Biostatistics, Sun Yat-Sen University, Guangzhou, China; ^5^Department of Genetics and Endocrinology, Guangzhou Women and Children's Medical Center, Guangzhou Medical University, Guangzhou, China

**Keywords:** hormone replace therapy, bone mineral density, body composition, turner syndrome, China

## Abstract

A longitudinal observational study was performed comparing BMD and body composition in Turner syndrome girls before and after 1 year of HRT treatment. Whole body BMD, femur neck BMD, total hip BMD, and lean mass were significantly increased, but there was no difference in fat mass, and lumbar spine BMD.

**Purpose:** Low bone mineral density (BMD) is one of the major health problems in Turner syndrome (TS) patients, and a certain percentage of TS girls are treated with hormone replacement therapy (HRT) to improve their BMD, among other health benefits. While it is generally accepted that HRT improves BMD and body composition in adolescent and young adult TS patients, studies of HRT in Chinese TS patients are limited.

**Methods:** To investigate the effects of HRT in Chinese TS girls, we performed a longitudinal observational study which compared measurement of BMD and body composition by dual energy X-ray absorptiometry (DXA) using a Lunar DXA densitometer in 20 Chinese adolescent and young adult TS patients (average age = 18) before and after 1 year of HRT treatment.

**Results:** Whole body BMD (0.85 vs. 0.87 g/cm^2^, *P* < 0.001), femur neck BMD (0.6 vs. 0.62 g/cm^2^, *P* = 0.02), total hip BMD (0.68 vs. 0.71 g/cm^2^, *P* = 0.003) and whole body lean mass (30.39 vs. 31.66 kg, *P* = 0.002) were significantly increased in these patients after 1 year HRT treatment, but there was no difference in whole body fat mass, android:gynoid ratio and lumbar spine BMD.

**Conclusions:** In summary, our study found that HRT was an effective way to increase whole body BMD, femur neck BMD, total hip BMD and whole body lean mass in Chinese TS girls, with no effect on whole body fat mass, android:gynoid ratio or lumbar spine BMD.

## Introduction

Turner syndrome (TS), a disease caused by the deletion or structural abnormality of one X chromosome, is characterized by short stature, low hormone, and congenital under development of the uterus and ovaries (1). Although the incidence of TS is between 1/2,500 and 1/3,000 among live-born girls, it is one of the most common chromosomal diseases in females (2). The symptoms of TS are varied, but all TS patients report a higher frequency of medical conditions compared to the normal population (3). For adolescent and young adult patients with TS, bone health and bone mineral density (BMD) are major concerns (4). TS women have a higher frequency of osteopenia/osteoporosis and bone fracture than normal females (5). The reasons for low BMD and increased bone fragility are multifarious, including chronic hormone deficiency (especially estrogen deficiency), X-chromosome abnormalities (especially haploinsufficiency of the SHOX gene), and other environmental factors (such as few/decreased physical activity due to skeletal muscular dysplasia) (2, 4, 6).

Hormone replacement therapy (HRT) is an important strategy for improving BMD in TS patients because chronic estrogen deficiency is one of the major reasons for bone loss in TS patients. However, several studies found that the BMD of TS patients with HRT were still very low (7, 8). One study indicated that lumbar spine BMD was increased after HRT, while other BMD parameters (e.g., hip BMD, forearm BMD and ultra-distal BMD) remained unchanged (9).

HRT should also have a positive effect on preventing body fat mass gain in women with malfunctioning ovaries, because the loss of endogenous estrogen may lead to fat mass gain in those women (10). However, whether HRT can change the body composition in TS patients remains unclear. It has previously been reported that whole body fat mass measured by dual energy X-ray absorptiometry (DXA), body mass index (BMI), and waist-to-hip ratio (WHR) were not changed by cyclical HRT in 9 non-obese TS patients (mean age: 23 years), but whole body lean mass measured by DXA had a tendency to increase (*p* = 0.054) after 1 year HRT treatment (11). Recently, a 5-year prospective randomized controlled clinical trial in Demark found that whole body fat mass did not change in the HRT group, but whole body lean mass increased in the high dose HRT group (Trisekvens with estradiol 2 mg on day 1–22 of the menstrual cycle) of young TS women (mean age: 19.2 years) (12).

To investigate the effects of HRT on BMD and body composition in Chinese adolescent and young adult TS patients, we performed this study by comparing BMD status at baseline with 1 year follow-up after HRT was started in TS patients. BMD and body composition were assessed by DXA which is the diagnostic gold standard tool for the diagnosis of osteopenia/osteoporosis and sarcopenia. To reduce bias caused by other BMD related factors including age, ethnicity, and ovarian function status, we performed this study among TS patients with primary amenorrhea. HRT is initiated at the age of 13 or above for most TS girls, therefore all participants were above 13 years of age and 19 of the 20 participants were over 15 years of age.

## Methods

### Participants

A total of 20 adolescent and young adult TS patients (including X0 and mosaicism, diagnosed based on the results of chromosome analysis, for details see Supplementary Table 1) with primary amenorrhea participated in the current study. For each subject, cytogenetic analysis was performed on peripheral blood lymphocytes according to standard Giemsa stain G banding technology with 350–450 bands, more than 30 cells were karyotyped per patient (13). Exclusion criteria for our study were other chronic bone diseases which may influence BMD (e.g., osteochondrodysplasia and malignant osteopetrosis), other diseases which may influence BMD (e.g., type 1 or type 2 diabetes, hyperthyroidism, coeliac disease, other thyroid disorders) or treatment with drugs associated with bone metabolism or BMD (e.g., glucocorticoid and growth hormone). Since the karyotype of TS patients is not associated with their BMD status and body composition (14, 15), the present study did not exclude TS patients with mosaicism. The participants were treated and followed by the same physicians in the Divisions of Pediatric Endocrinology and Gynecology Endocrinology of Guangzhou Medical University, Guangzhou Women and Children's Medical Center, China. The baseline clinical data, including age, weight, height, body mass index (BMI), whole body fat mass, whole body lean mass, whole body mass, android:gynoid ratio, whole body BMD, lumbar spine BMD, femur neck BMD, and total hip BMD are shown in Table 1. All of the participants were then started on HRT, and measurements repeated at 6 and 12 months post starting treatment. There was only one patient <15 years old who was started with continuous low-dose estradiol valerate therapy (0.5 mg daily for the first 6 months, continued with 1 mg daily for another 6 months; Progynova), the other participants who were over 15 years of age received cyclic HRT (17β-estradiol 2 mg/d for 28 days, adding dydrogesterone 10 mg/day for 14 days on day 14; Fenmatong) (16). Pubertal/sexual maturity was assessed following Marshall-Tanner criteria (17, 18). The study was performed in accordance with institutional guidelines, and written informed consent was obtained from the participants enrolled or the parents of those with a chronological age below 18 years. The study was approved by the ethics committee for human investigation at Guangzhou Women and Children's Medical Center.

**Table 1:** Basic clinical data of adolescent and young adult TS patients.

**Variables**	**Mean (Range)**	**SD**
Age	18.45(16–21)	3.07
Weight (Kg)	44.64(32.5–65)	10.09
Height (cm)	144.89(131.8–163)	8.77
BMI (Kg/m^2^)	21.30(16.5–21.4)	4.65
Whole Body BMD (g/cm^2^)	0.85(0.77–0.96)	0.07
Lumbar Spine BMD (g/cm^2^)	0.69(0.57–0.79)	0.09
Femur Neck BMD (g/cm^2^)	0.60(0.44–0.82)	0.1
Total Hip BMD (g/cm^2^)	0.68(0.52–0.90)	0.09
Whole Body Fat Mass (Kg)	15.59(8.26–27.16)	5.30
Whole Body Lean Mass (Kg)	30.39(23.85–39.04)	4.55
Whole Body Mass (Kg)	45.98(32.11–66.19)	8.99

*BMI, body mass index; BMD, bone mineral density; SD, Standard Deviation*.

### BMD Evaluation

Whole body fat mass, whole body lean mass, whole body mass, android:gynoid ratio, and BMD in different sites were measured by dual energy X-ray absorptiometry (DXA) using a Lunar DXA densitometer (Lunar Corporation, Madison WI, U.S.A.). The DXA device is composed of a set of equipment including a super stable X-ray generator, a computer and data analysis software. The X-rays are emitted by the X-ray generator, which can penetrate the body. Different tissues have different attenuation signals of X-ray due to different thickness and density. After processing by computer software, the content of different tissues is calculated. To date, determination of bone mineral density by DXA is the gold standard for the diagnosis of osteopenia/osteoporosis and sarcopenia (19, 20). In addition, DXA determination of whole body fat mass and whole body lean mass has a high sensitivity and specificity. The low radiation dose of DXA also ensures its safety in adolescents and young adults. The BMD measurements were conducted before HRT therapy, 6 months after HRT therapy and 1 year after HRT therapy.

### Statistical Analysis

Basic statistics including means and standard deviation (SD) were computed by SPSS (Statistical Package of Social Sciences, Chicago, IL, USA) for Windows software program version 19.0. Differences between baseline and that of follow-up after HRT were tested by ANOVA for Repeated Measurement Data. Results are presented as a mean±standard deviation (SD). A *P* < 0.05 was considered as the threshold for nominal significance.

## Results

The basic clinical data of the 20 TS patients is shown in Table 1. Of the participants, the mean age is 18, the mean BMI is 21.3 Kg/m^2^, the mean whole body fat mass is 15.59 Kg, the mean whole body lean mass is 30.38 Kg, the mean whole body mass is 45.98 Kg, the mean whole body BMD is 0.85 g/cm^2^, the mean lumbar spine BMD is 0.69 g/cm^2^, the mean femur neck BMD is 0.60 g/cm^2^, and the mean total hip BMD is 0.68 g/cm^2^. For reference, data for normal 18 year old girls is shown in Supplementary Table 1. The karyotype and Tanner stage of puberty for each of the study participants is shown in Supplementary Table 2.

Since there were two follow-up points and some missing data in this study, we used a linear mixed effect model of ANOVA for repeated measurement data. The first step was to analyse the trends of each measurement index in the follow-up period. There was a significant difference in whole body BMD (*P* < 0.0001), whole body BMC (Bone Mineral Content, *P* < 0.0001), whole body bone area (*P* = 0.003), femur neck BMD (*P* = 0.01), total hip BMD (*P* = 0.002), total hip BMC (*P* = 0.05), whole body lean mass (*P* = 0.004), and whole body mass (*P* = 0.005) (Table 2). However, there was no significant difference found in lumbar spine BMD, lumbar spine BMC, lumbar spine bone area, femur neck BMC, femur neck bone area, total hip bone area, whole body fat mass, and android:gynoid ratio.

**Table 2:** Change of bone mineral status and body composition after 1 year HRT.

**Variables**	**Mean (Range)**	**SD**	***P*-Value**
Whole Body BMD	0.86 (0.78–0.94)	0.06	<0.0001
Whole Body BMC	1313.49 (1054.15–1795.19)	181.03	<0.0001
Whole Body Bone Area	1521.58 (1335.39–1800.66)	127.16	0.003
Lumbar Spine BMD	0.73 (0.6–0.86)	0.07	0.1
Lumbar Spine BMC	33.80 (21.85–46.00)	7.38	0.6
Lumbar Spine Bone Area	46.39 (36.22-56.33)	23.63	0.9
Femur Neck BMD	0.62 (0.64–0.86)	0.11	0.01
Femur Neck BMC	2.79 (2.18–3.90)	0.47	0.05
Femur Neck Bone Area	4.53 (3.40–6.04)	0.58	0.8
Total Hip BMD	0.70 (0.55–0.93)	0.10	0.002
Total Hip BMC	19.90 (16.23–26.85)	2.70	0.05
Total Hip Bone Area	28.48 (25.14–34.14)	2.55	0.8
Whole Body Fat Mass	17521.74 (8676.63–29379.22)	5677.88	0.1
Whole Body Lean Mass	31959.37 (24855.73–40769.51)	4424.01	0.0002
Whole Body Mass	49481.11 (33532.36–69764.00)	9350.11	0.005
Height	147.46(138.5–163)	6.03	0.1

*BMD, bone mineral density; BMC, bone mineral content; SD, Standard Deviation*.

**Figure 1:**
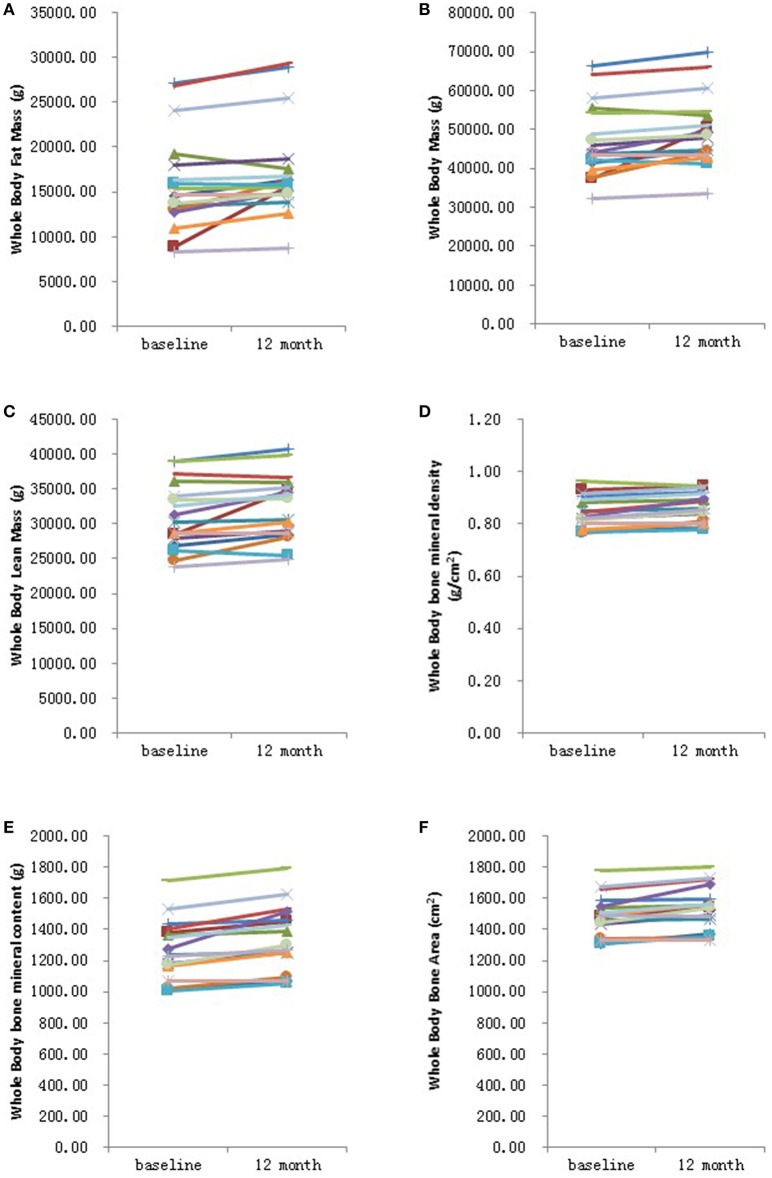
Graphical representation in changes in whole body measurements before and after 12 months HRT. **(A)** Whole body fat mass; **(B)** Whole body mass; **(C)** Whole body lean mass; **(D)** Whole body bone mineral density; **(E)** Whole body bone mineral content; **(F)** Whole bone area. *N* = 17.

**Figure 2:**
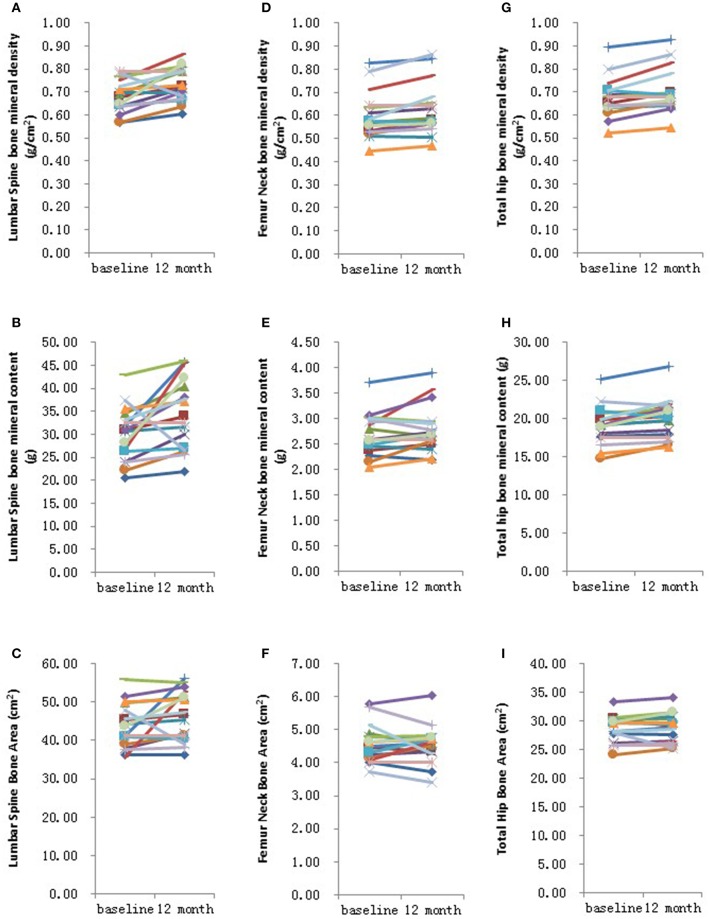
Graphical representation in changes in individual site measurements before and after 12 months HRT. **(A)** Lumbar spine bone mineral density; **(B)** Lumbar spine bone mineral content; **(C)** Lumbar spine bone area; **(D)** Femur neck bone mineral density; **(E)** Femur neck bone mineral content; **(F)** Femur neck bone area; **(G)** Total hip bone mineral density; **(H)** Total hip bone mineral content; **(I)** Total hip bone area. *N* = 17.

Further analysis showed that after 6 months HRT treatment, whole body BMD (*P* = 0.01), whole body lean mass (*P* = 0.004) and whole body mass (*P* = 0.04) were significantly increased, but there was no significant difference found in the BMD of other sites (Supplementary Table 3; Supplementary Figures 1, 2). After 12 months HRT treatment, whole body BMD (*P* < 0.001), whole body BMC (*P* < 0.001), whole body bone area (*P* < 0.001), femur neck BMD (*P* = 0.02), total hip BMD (*P* = 0.003), total hip BMC (*P* = 0.04), whole body lean mass (*P* = 0.002), and whole body mass (*P* = 0.007) were significantly increased, and there was no significant difference found in lumbar spine BMD, lumbar spine BMC, lumbar spine bone area, femur neck bone area and total hip bone area (Table 3; Figures 1, 2).

**Table 3:** Bone mineral status and body composition baseline and after 12 months HRT.

	**Baseline (*n* = 20)**	**12 months (*n* = 17)**	***P*-Value**
Whole Body Fat Mass (g)	15594.76 ± 5302.42	16874.49 ± 5869.95	0.04
Whole Body Lean Mass (g)	30387.51 ± 4549.32	31657.94 ± 4283.18	0.002
Whole Body Mass (g)	45982.27 ± 8991.47	48532.43 ± 9558.27	0.007
Android:Gynoid Ratio	0.92 ± 0.13	0.93 ± 0.13	0.2
Whole Body BMD (g/cm^2^)	0.85 ± 0.07	0.87 ± 0.07	0.0001
Whole Body BMC (g)	1253.86 ± 192.02	1301.21 ± 156.58	0.0001
Whole Body Bone Area (cm^2^)	1470.26 ± 131.35	1491.87 ± 108.24	0.0001
Lumbar Spine BMD (g/cm^2^)	0.69 ± 0.09	0.73 ± 0.09	0.1
Lumbar Spine BMC (g)	30.66 ± 6.57	32.73 ± 7.09	0.2
Lumbar Spine Area (cm^2^)	43.96 ± 5.42	44.42 ± 5.61	0.4
Femur Neck BMD (g/cm^2^)	0.6 ± 0.1	0.62 ± 0.11	0.02
Femur Neck BMC (g)	2.62 ± 0.4	2.7 ± 0.39	0.03
Femur Neck Bone Area (cm^2^)	4.4 ± 0.63	4.37 ± 0.51	0.5
Total Hip BMD (g/cm^2^)	0.68 ± 0.09	0.71 ± 0.1	0.003
Total Hip BMC (g)	18.77 ± 2.49	19.32 ± 2.55	0.04
Total Hip Area (cm^2^)	27.71 ± 2.99	27.35 ± 2.78	0.7

*BMD, bone mineral density; BMC, bone mineral content*.

## Discussion

In summary, our results suggest that HRT treatment in adolescent and young adult TS patients is generally effective in improving BMD, especially for whole body BMD, hip BMD and femoral neck BMD. However, it must be noted that while there was an improvement for all the patients their BMD was still below that considered normal for females of their age (data not shown). In contrast to a previous study (9), we did not observe an improvement in lumbar spine BMD which may in part be due to the small sample size of the current study. At the same time, whole body lean mass and whole body mass were significantly enhanced, while the whole body fat mass and android:gynoid ratio did not change.

For TS patients, low BMD and increased risk of bone fractures are two major bone health concerns. Across all age ranges, TS patients have lower BMD compared to age matched normal control girls and women (4, 21, 22). Low BMD during climacterium and senectitude is an important risk factor for bone fractures, therefore it is essential to improve their BMD not only during climacterium and senectitude but also in adolescent and young ages.

In general, HRT was thought to be an important option for improving BMD in TS osteopenia or osteoporotic women because estrogen-deficiency and elevated follicle stimulating hormone (FSH) levels are two of the major reasons for bone loss in TS individuals (4, 23). In those estrogen-deficient and elevated FSH patients, the increasing level of serum estrogen not only increases the number of active osteoblasts but also decreases the high bone turnover rate (24). One previous study demonstrated that low BMD in TS patients was related to high osteoclastogenesis, which in turn was caused by high levels of FSH (23). HRT is an effective way to reduce FSH levels, which may help to improve BMD in TS patients. However, several studies have reported that the BMD of TS patients after HRT are still very low (7, 8).

Some differences in these different studies may be explained by the use of different HRT protocols in different centers. Exogenous E2 can be administered various different routes including transdermally (TD), orally, transvaginally, or by injection. TD administration by-passes the liver, there are no efficacy studies on age at onset and duration of treatment, furthermore, TD is not available in our hospital. Oral estrogens include 17β-estradiol, estradiol valerate and conjugated estrogens (CEE). Previous studies have found no significant differences in fasting insulin concentration, protein turnover, lipolysis, BMI or waist-to-hip ratio between groups with TD vs. oral natural estrogen treatment (25–27). CEE's raise blood pressure and are therefore not recommended. All but three participants were not sexually active, so transvaginal administration was not recommended for TS girls in our study. Administration by injection is not preferred by most patients. Therefore, oral administration was used for the current study. It is recommended that in girls low dose estrogen be given to induce puberty (2–3 years), at which point estrogen should be increased to 2 mg/d and progesterone added. However, GWCMC only has 2 mg 17β-estradiol and because the patients were older than 15 years they were automatically started on cyclic HRT, all the patients subsequently started breakthrough bleeding after treatment was started.

In the present study, we found that whole body BMD, femur neck BMD and total hip BMD were significantly increased after HRT was given among adolescent and young adult TS patients, but there was no difference in lumbar spine BMD. Our results indicated that the mechanism of action of HRT in improving BMD varies depending on the skeletal site being assessed. For whole body BMD, femur neck BMD and total hip BMD, chronic estrogen deficiency may be the major reason for bone loss in these sites. But for lumbar spine BMD, X-chromosome abnormalities, or other genetic factors may be the major reason for bone loss in adolescent and young adult TS patients (14). Our findings also demonstrated that whole body BMD was the earliest site among the enhanced BMD sites, which was increased after 6 months HRT. Femur neck BMD and total hip BMD were significantly increased after 1 year HRT, but total hip bone area did not alter. This finding implies that HRT may increase bone mineral content but not its size. Whole body BMD increased after 6 months HRT and whole body bone area increased after 1 year HRT, which suggests that the increase in bone mineral content precedes an increase in bone size after HRT among adolescent and young adult Turner syndrome patients.

Some studies have also reported an effect of HRT on body composition including fat mass and lean mass in TS patients, although this finding has not been consistent (10–12). The loss of endogenous estrogen may lead to fat mass gain so that TS women were found to have higher fat mass and higher BMI than age matched control women (28). The current study reports that HRT increased whole body lean mass and whole body mass, while the whole body fat mass and android:gynoid ratio were not altered. This finding suggests that HRT has a positive effect on body composition, enhanced lean mass improves muscle strength and can prevent bone fractures (4). In addition, keeping a fit body shape and low fat mass can also help prevent other serious chronic metabolic health problems such as obesity, type 2 diabetes, and cardiovascular disease for TS patients. Therefore, the positive effect of HRT on body composition in TS women may also lead to lifelong health benefits.

The current study demonstrates that HRT was an effective way to increase whole body BMD, femur neck BMD, total hip BMD, but not lumbar spine BMD in Chinese TS girls, which indicated that for whole body BMD, femur neck BMD and total hip BMD, chronic estrogen deficiency may be the major reason for bone loss in these sites, but for lumbar spine BMD, X-chromosome abnormalities or other genetic factors may be the major reason for bone loss in Chinese adolescent and young adult TS patients. In addition, HRT was an effective way to enhance whole body lean mass and whole body mass in TS girls, and it has no effect on whole body fat mass and android:gynoid ratio. However, it must be noted that due to the rarity of this condition the sample size in the current study was small and larger studies are required. Our present study is a 1-year longitudinal study, which only shows the effect of HRT on BMD and body composition at this time point. Further long-term follow-up studies are needed to evaluate the long-term efficacy of HRT and determine the peak BMD after HRT for TS patients during their life time. In conclusion, our findings suggest that HRT has a significant positive impact on increasing BMD, improving muscle strength, preventing bone fracture and keeping a fitness body shape, which will give TS women lifelong health benefits.

## Data Availability

All datasets generated for this study are included in the manuscript and/or the Supplementary Files.

## Ethics Statement

The study was performed in accordance with institutional guidelines, and written informed consent was obtained from the participants enrolled or the parents of those with a chronological age below 18 years. The study was approved by the ethics committee for human investigation at Guangzhou Women and Children's Medical Center.

## Author Contributions

LLi and XQ designed and prepared the draft of manuscript. LLi and LLiu conceived the idea, supervised all research. GL revised the manuscript. LY analyzed the data. ZL participated in the experiments. All authors reviewed the manuscript.

### Conflict of Interest Statement

The authors declare that the research was conducted in the absence of any commercial or financial relationships that could be construed as a potential conflict of interest.

## Supplementary Material

Supplementary Table 1 Basic clinical data of normal 18-year-old girls.

Supplementary Table 2 Change of bone mineral status and body composition after 6 months HRT.

Supplementary Table 3 Summary of genotype and change of tanner stage after 1year HRT.

Supplementary Figure 1 Graphical representation in changes in whole body measurements before and after 6 months HRT. **(A)** Whole body fat mass; **(B)** Whole body mass; **(C)** Whole body lean mass; **(D)** Whole body bone mineral density; **(E)** Whole body bone mineral content; **(F)** Whole bone area. *N* = 10.

Supplementary Figure 2 Graphical representation in changes in individual site measurements before and after 6 months HRT. **(A)** Lumbar spine bone mineral density; **(B)** Lumbar spine bone mineral content; **(C)** Lumbar spine bone area; **(D)** Femur neck bone mineral density; **(E)** Femur neck bone mineral content; **(F)** Femur neck bone area; **(G)** Total hip bone mineral density; **(H)** Total hip bone mineral content; **(I)** Total hip bone area. *N* = 10.

## Abbreviations

BMD: bone mineral density
TS: Turner syndrome
HRT: Hormone replacement therapy
DXA: dual energy X-ray absorptiometry
BMI: Body mass index
WHR: waist-to-hip ratio
BMC: Bone mineral content
FSH: Follicle stimulating hormone.

## References

[1] Ari M Bakalov VK Hill S Bondy CA. The effects of growth hormone treatment on bone mineral density and body composition in girls with turner syndrome. J Clin Endocrinol Metabol. (2006) 91:4302–5. 10.1210/jc.2006-1351 16940444

[2] Sybert VP McCauley E. Turner's syndrome. New Engl J Med. (2004) 351:1227–38. 10.1056/NEJMra030360 15371580

[3] Naess EE Bahr D Gravholt CH. Health status in women with Turner syndrome: a questionnaire study on health status, education, work participation and aspects of sexual functioning. Clin Endocrinol. (2010) 72:678–84. 10.1111/j.1365-2265.2009.03715.x 19769615

[4] Bakalov VK Bondy CA. Fracture risk and bone mineral density in Turner syndrome. Rev Endocr Metab Disord. (2008) 9:145–51. 10.1007/s11154-008-9076-2 18415020

[5] Tsuburai T Nakamura T Yoshikata H Miyagi E Sakakibara H. Eldecalcitol increases bone mass in patients with Turner syndrome who have insufficient bone mass acquisition after estrogen replacement therapy. Endocr J. (2018) 65:629–38. 10.1507/endocrj.EJ17-0498 29607913

[6] Ross JL Kowal K Quigley CA Blum WF Cutler GB Crowe B. The phenotype of short stature homeobox gene (SHOX) deficiency in childhood: contrasting children with leri-weill dyschondrosteosis and turner syndrome. J Pediatr. (2005) 147:499–507. 10.1016/j.jpeds.2005.04.069 16227037

[7] Lanes R Gunczler P Esaa S Martinis R Villaroel O Weisinger JR. Decreased bone mass despite long-term estrogen replacement therapy in young women with Turner's syndrome and previously normal bone density. Fertil Steril. (1999) 72:896–9. 10.1016/S0015-0282(99)00389-1 10560996

[8] Högler W Briody J Moore B Garnett S Lu PW Cowell CT. Importance of estrogen on bone health in turner syndrome: a cross-sectional and longitudinal study using dual-energy X-ray absorptiometry. J Clin Endocrinol Metab. (2004) 89:193–9. 10.1210/jc.2003-030799 14715849

[9] Cleemann L Hjerrild BE Lauridsen AL Heickendorff L Christiansen JS Mosekilde L. Long-term hormone replacement therapy preserves bone mineral density in Turner syndrome. Eur J Endocrinol. (2009) 161:251–7. 10.1530/EJE-09-0020 19447901

[10] Monda V Salerno M Fiorenzo M Villano I Viggiano A Sessa F. Role of sex hormones in the control of vegetative and metabolic functions of middle-aged women. Front Physiol. (2017) 8:773. 10.3389/fphys.2017.00773 29046646 PMC5632804

[11] Nielsen K Abildgaard JS. The development and validation of a job crafting measure for use with blue-collar workers. Work Stress. (2012) 26:365–84. 10.1080/02678373.2012.733543 23236220 PMC3516817

[12] Cleemann L Holm K Kobbernagel H Kristensen B Skouby SO Jensen AK. Dosage of estradiol, bone and body composition in Turner syndrome: a 5-year randomized controlled clinical trial. Eur J Endocrinol. (2017) 176:233–42. 10.1530/EJE-16-0582 27881458

[13] Li L Li Q Wang Q Liu L Li R Liu H. Rare copy number variants in the genome of Chinese female children and adolescents with Turner syndrome. Bioscience Rep. (2019) 39:BSR20181305. 10.1042/BSR20181305 30530863 PMC6328875

[14] El-Mansoury M Barrenäs M Bryman I Hanson C Larsson C Wilhelmsen L. Chromosomal mosaicism mitigates stigmata and cardiovascular risk factors in Turner syndrome. Clin Endocrinol. (2007) 66:744–51. 10.1111/j.1365-2265.2007.02807.x 17381484

[15] Shi K Liu L He Y Li D Yuan L Lash GE. Body composition and bone mineral status in patients with Turner syndrome. Sci. Rep. (2016) 6: 38026. 10.1038/srep38026 27901060 PMC5128814

[16] Stein BR Thomas VA Lorentz LJ Strahm BD Predicting macronutrient concentrations from loblolly pine leaf reflectance across local and regional scales. Gisci Remote Sens. (2014) 51:269–87. 10.1080/15481603.2014.912875

[17] Marshall WA Tanner JM. Variations in the pattern of pubertal changes in boys. Arch Dis Child. (1970) 45:13–23. 10.1136/adc.45.239.13 5440182 PMC2020414

[18] Marshall WA Tanner JM. Variations in pattern of pubertal changes in girls. Arch Dis Child. (1969) 44:291–303. 10.1136/adc.44.235.291 5785179 PMC2020314

[19] Cruz-Jentoft AJ Baeyens JP Bauer JM Boirie Y Cederholm T Landi F. Sarcopenia: European consensus on definition and diagnosis: report of the european working group on sarcopenia in older people. Age Ageing. (2010) 39:412–23. 10.1093/ageing/afq034 20392703 PMC2886201

[20] Osteoporosis prevention diagnosis and therapy NIH Consensus Statement. (2000) 17:1–45. 11525451

[21] Pitukcheewanont P Numbenjapon N Safani D Rossmiller S Gilsanz V Costin G. Bone size and density measurements in prepubertal children with Turner syndrome prior to growth hormone therapy. Osteoporosis Int. (2011) 22:1709–15. 10.1007/s00198-010-1375-2 20827549

[22] Soucek O Schönau E Lebl J Willnecker J Hlavka Z Sumnik Z. A 6-year follow-up of fracture incidence and volumetric bone mineral density development in girls with turner syndrome. J Clin Endocrinol Metab. (2018) 103:1188–97. 10.1210/jc.2017-02381 29300907

[23] Faienza MF Brunetti G Ventura A Piacente L Messina MF De Luca F. Mechanisms of enhanced osteoclastogenesis in girls and young women with Turner's Syndrome. Bone. (2015) 81:228–36. 10.1016/j.bone.2015.07.021 26208797

[24] Levin VA Jiang X Kagan R. Estrogen therapy for osteoporosis in the modern era. Osteoporosis Int. (2018) 29:1049–55. 10.1007/s00198-018-4414-z 29520604

[25] Mauras N Shulman D Hsiang HY Balagopal P Welch S. Metabolic effects of oral versus transdermal estrogen in growth hormone-treated girls with turner syndrome. J Clin Endocrinol Metab. (2007) 92:4154–60. 10.1210/jc.2007-0671 17711924

[26] Alves STDF Gallichio CT Guimaraes MM. Insulin resistance and body composition in Turner syndrome: effect of sequential change in the route of estrogen administration. Gynecol Endocrinol. (2006) 22:590–4. 10.1080/08916930600929586 17135039

[27] Reinehr T Lindberg A Toschke C Cara J Chrysis D Camacho-Hubner C. Weight gain in Turner Syndrome: association to puberty induction?—longitudinal analysis of KIGS data. Clin Endocrinol. (2016) 85:85–91. 10.1111/cen.13044 26921881

[28] Whitmarsh T Otake Y Uemura K Takao M Sugano N Sato Y. A cross-sectional study on the age-related cortical and trabecular bone changes at the femoral head in elderly female hip fracture patients. Sci. Rep. (2019) 9:305. 10.1038/s41598-018-36299-y 30670734 PMC6343024

