# Supplementary material for: Effect of Hormone Replacement Therapy on Bone Mineral Density and Body Composition in Chinese Adolescent and Young Adult Turner Syndrome Patients
Source: Front Endocrinol (Lausanne). 2019 Jun 12;10:377. doi: 10.3389/fendo.2019.00377 (PMC6582219; doi:10.3389/fendo.2019.00377)
Supplement: Supplementary Table 1 — Basic clinical data of normal 18-year-old girls. [file Table_1.DOCX]

**Supplementary Table 1: Basic clinical data of normal 18-year-old girls**

| Variables | Mean | Std. Deviation |
| --- | --- | --- |
| Age | 18.30 | 0.30 |
| Weight (Kg) | 48.6 | 6.60 |
| Height (cm) | 157.5 | 5.30 |
| BMI (Kg/m^2^) | 19.5 | 2.00 |
| Whole Body BMD (g/cm^2^) | 1.059 | 0.083 |
| Whole Body Fat Mass (Kg) | 12.90 | 3.40 |
| Whole Body Lean Mass (Kg) | 32.20 | 4.90 |
|  |  |  |

**Supplementary Table2:** **Change of bone mineral status and body composition after 6 months HRT**

| Variables | Mean (Range) | Std. Deviation |
| --- | --- | --- |
| Whole Body BMD | 0.86 （0.78-0.97） | 0.06 |
| Whole Body BMC | 1324.31 （1013.35-1688.61） | 203.01 |
| Whole Body Bone Area | 1528.20 （1290.84-1737.56） | 144.19 |
| Lumbar Spine BMD | 0.69 （0.60-0.82） | 0.07 |
| Lumbar Spine BMC | 31.11 （22.91-42.60） | 6.02 |
| Lumbar Spine Bone Area | 44.78 （36.84-53.17） | 5.84 |
| Femur Neck BMD | 0.61 （0.51-0.81） | 0.09 |
| Femur Neck BMC | 2.80 (2.33-3.57） | 0.46 |
| Femur Neck Bone Area | 4.59 (3.98-5.95) | 0.53 |
| Total Hip BMD | 0.69 (0.58-0.90) | 0.09 |
| Total Hip BMC | 19.58 （15.41-25.08） | 2.57 |
| Total Hip Bone Area | 0.69 （24.39-33.89） | 0.07 |
| Whole Body Fat Mass | 18062.24 （13211.92-30712.60） | 6180.59 |
| Whole Body Lean Mass | 33428.04 （25894.14-40502.29） | 5174.02 |
| Whole Body Mass | 51490.28 （40270.19-68870.41） | 10175.87 |
|  |  |  |

**Supplementary Table 3 Summary of genotype and change of tanner stage after 1year HRT**

| Patient NO. | Genotype | tanner stage breast | | tanner stage pubic hair | |
| --- | --- | --- | --- | --- | --- |
|  |  | Baseline | 12 month | baseline | 12 month |
| Patient 1 | 45,X | II | III | I | II |
| Patient 2 | 45,X | II | IV | I | III |
| Patient 3 | 45,X | II | III | II | III |
| Patient 4 | 45,X[19]/46,XX[81] | II | III | II | III |
| Patient 5 | 45,X | II | III | II | III |
| Patient 6 | 45,X | II | IV | II | IV |
| Patient 7 | 45,X[70]/46,X,+mar30) | III | IV | II | III |
| Patient 8 | 45,X[65]/47,XXX[35] | II | IV | II | III |
| Patient 9 | 46,x,del(X)(q22.1) | III | IV | III | IV |
| Patient 10 | 46,x,del(X)(q22.1) | III | IV | III | IV |
| Patient 11 | 47x,+mar | II | III | II | III |
| Patient 12 | 46，X，i(X)(q10) | II | III | II | IV |
| Patient 13 | 45,X | III | V | II | IV |
| Patient 14 | 45,X | II | III | II | III |
| Patient 15 | 45,X | II | IV | II | III |
| Patient 16 | 45,X | II | III | II | III |
| Patient 17 | 45,X | II | IV | II | IV |
