# Supplementary figures and images for: Effect of Hormone Replacement Therapy on Bone Mineral Density and Body Composition in Chinese Adolescent and Young Adult Turner Syndrome Patients
Source: Front Endocrinol (Lausanne). 2019 Jun 12;10:377. doi: 10.3389/fendo.2019.00377 (PMC6582219; doi:10.3389/fendo.2019.00377)

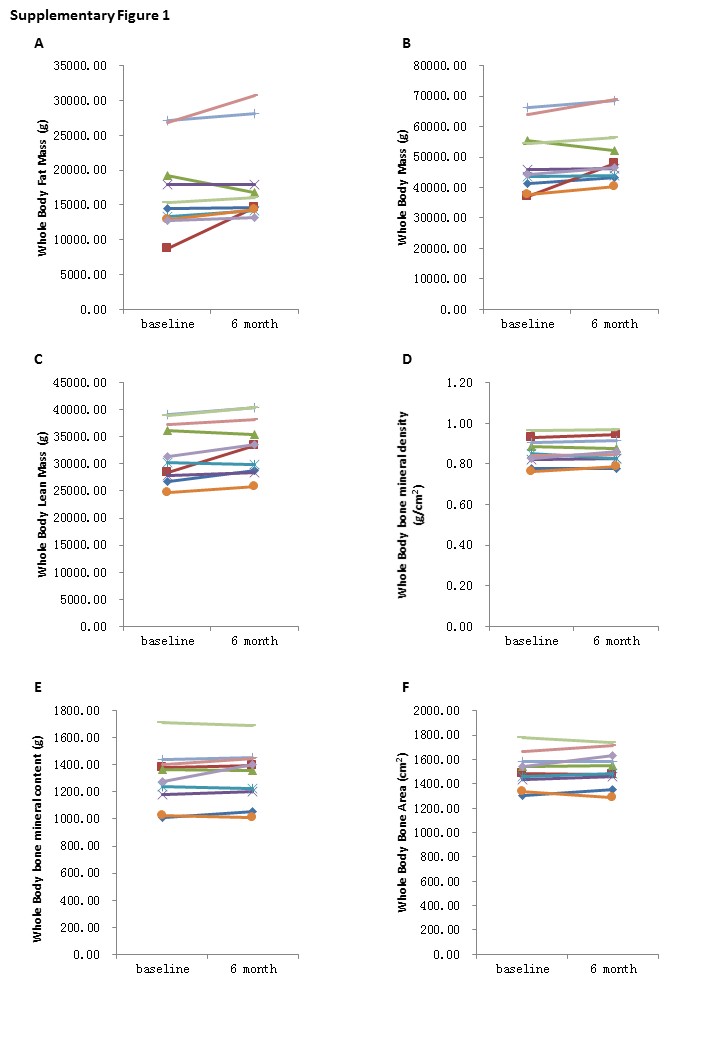

Supplement: Supplementary Figure 1 — Graphical representation in changes in whole body measurements before and after 6 months HRT. (A) Whole body fat mass; (B) Whole body mass; (C) Whole body lean mass; (D) Whole body bone mineral density; (E) Whole body bone mineral content; (F) Whole bone area. N = 10. [file Image_1.jpeg]

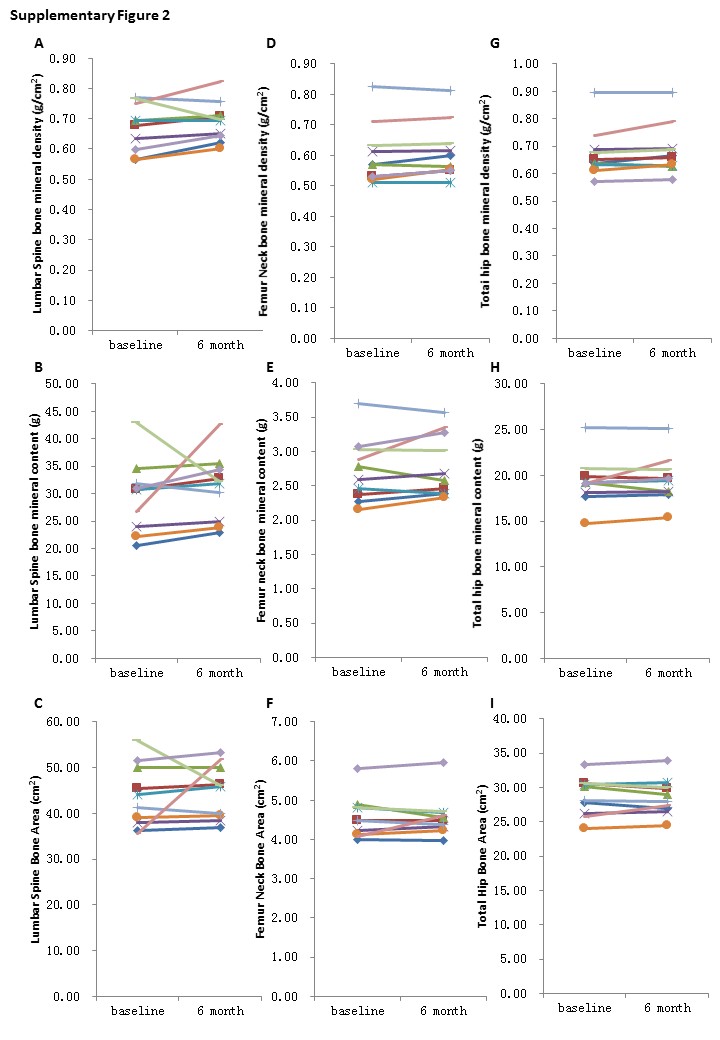

Supplement: Supplementary Figure 2 — Graphical representation in changes in individual site measurements before and after 6 months HRT. (A) Lumbar spine bone mineral density; (B) Lumbar spine bone mineral content; (C) Lumbar spine bone area; (D) Femur neck bone mineral density; (E) Femur neck bone mineral content; (F) Femur neck bone area; (G) Total hip bone mineral density; (H) Total hip bone mineral content; (I) Total hip bone area. N = 10. [file Image_2.jpeg]
